# Effects of vegetation cover and slope on soil erosion in the Eastern Chinese Loess Plateau under different rainfall regimes

**DOI:** 10.7717/peerj.11226

**Published:** 2021-04-12

**Authors:** Congjian Sun, Huixin Hou, Wei Chen

**Affiliations:** School of Geographical Science, Shanxi Normal University, Linfen, China

**Keywords:** Erosive precipitation, Vegetation measures, Soil conservation, Water conservation, Eastern Chinese Loess Plateau

## Abstract

Soil erosion is a critical environmental problem of the Chinese Loess Plateau (CLP). The effects of vegetation cover on soil erosion reduction under different rainfall types are not well understood especially in the eastern Chinese Loess Plateau (ECLP). In this study, we monitored runoff and sediment yield at the Fengjiagou water and soil conservation station with five types of vegetation cover (arbor trees (ARC), shrubs (SHC), arable (ABC), natural vegetation (NVC), and artificial grass (APC)) and three slope gradients (10°, 15°, and 20°) in the ECLP. Based on long-term monitoring data, five rainfall types were classified by the maximum 30 min rainfall intensity (I30). We also quantitatively revealed the interactive effects of different types precipitation, vegetation cover and slope gradients on regional soil erosion. The results showed that (1) The RII (13 times) and RIII (eight times) type are the most threatening erosive rainfall in this region. (2) The ARC and SHC type were most beneficial for soil and water conservation in the ECLP; The APC and ABC are not conductive to the prevention of regional soil erosion. (3) Runoff and sediment yields increased with the slope gradient. The farmland is vulnerable to soil erosion when the slope gradient exceeds 10°. The results of this study can improve the understanding of regional soil erosion processes on the ECLP and provide useful information for managing regional water and land resources.

## Introduction

The correct use of soil, one of the not renewable resources involved in human activities, affects the long-term sustainability of agricultural systems and ecosystems (Biddoccu et al., 2013; Biddoccu et al., 2018; Bagagiolo et al., 2018). Soil erosion is a major threat to our terrestrial ecosystems and a worldwide critical environmental problem (Sun, Shao & Liu, 2013). It directly causes soil deterioration and decreases land productivity (Zhou et al., 2016). At present, about more than 25 million km^2^ of land worldwide under the threat of soil erosion (Wang et al., 2019). Soil erosion has become an environmental problem that demands urgent attention from governmental management teams and requires reasonable regional control measures.

Research on regional soil erosion began in the mid-18th century (Fu et al., 2011). Previous studies have described the process of soil erosion in different regions in detail based on the soil erosion model, the method of indoor artificial rainfall simulations and the field plot observation method. For example, Giang, Giang & Toshiki (2017) simulated the response of soil erosion of the Southeast Asia to climate change and discovered that soil erosion increased significantly from August to October. In Europe, Cerdan et al. (2010) demonstrated that land use patterns heavily influenced the rate and quantity of soil erosion based on field observations and visible-near infrared (vis-NIR) spectroscopy data. Bagagiolo et al. (2018) systematically assessed soil erosion processes in vineyards in the NW Italy according to a long time series of erosive events records. Peña Angulo et al. (2020) evaluated the relationship of weather types on the seasonal and spatial variability of rainfall, runoff and sediment yield in the Western Mediterranean Basin. In Australia, Teng, Rossel & Shi (2016) estimated the factors controlling the revised universal soil loss equation (RUSLE) and modeled soil loss caused by soil erosion process. These studies have improved the overall understanding of soil erosion processes. Recently, systematically comprehending the interactive effects of main influencing factors on soil erosion is considered a key step to conserve soil and water. More and more researchers began to focus on the influencing factors of regional soil erosion and indicated that soil properties (Ritsema, 2003; Liu et al., 2012; Cerdan et al., 2010), vegetation coverage (Zhang, Tang & Zhang, 2009; Fattet et al., 2011; Zhang et al., 2019a; Zhang et al., 2019b), water content and of vehicle pass (Capello et al., 2019), and the weather type (Peña Angulo et al., 2020), slope gradient (Koulouri & Giourga, 2007; Sajikumar & Remya, 2015; Komatsu et al., 2018), and rainfall type (including intensity and duration) (Zhang et al., 2011; Fang et al., 2012) are the primary controlling factors for regional soil erosion processes. In China, soil erosion has become the most critical important environmental issue that restricts regional ecological security (Huang et al., 2010). There are serious soil erosion problems exist in the red earth region of southern China (Wang, Zhu & Wu, 2017), the black soil area of northeastern China (Liu et al., 2008), the purplish soil areas of southwestern China (Ma, Zhang & Wang, 2017), the karst slope regions of southwestern China (Peng & Wang, 2012), and the Chinese Loess Plateau (CLP) (Sun, Shao & Liu, 2013; Zhang et al., 2016; Zhang et al., 2019a; Zhang et al., 2019b). Focus on the sloping cultivated soils in hilly (such as loess, purple soil, red earth and weathered granite areas), numbers of research progress have promoted the development of regional soil and water conservation. Wang, Zhu & Wu (2017) analyzed the infiltration and soil erosion processes in the red earth regions of China to mitigate soil erosion and protect the surface soil. By comparing the soil erodibility and critical shear stress, Xing et al. (2018) studied the differences of rill erosion behaviors between loess and purple soils. Deng et al. (2019) compared the characteristics of runoff and sediment yield under different slope gradients and rainfall intensities for two kinds of different hillslopes with weathered granite and with exposed soils respectively based on the method of indoor artificial rainfall simulations.

As one of the most severely soil erosion regions in the world, the serious soil erosion of CLP not only leads to the decline of soil fertility but also results in regional environmental pollution (Ritsema, 2003; Ramos-Scharrón & Figueroa-Sánchez, 2017). In the last 50 years, the meteorological data shown the climate of CLP had an obvious tendency of warming and drying (Sun et al., 2020). However, the decreased rainfall did not reduce the soil loss in this region, while the soil erosion increased continuously, which was mainly due to the lower density of vegetation cover for lacking of enough water and human destruction. Since 1998, the Chinese government launched the Grain-to-Green Program, which has been mainly directed to soil and water conservation on the CLP (Zhao et al., 2013; Zhang et al., 2015). Although, the regional ecological environment has been improved after nearly 20 years of vegetation restoration (Fu, Zhang & Wang, 2016), the effect of land sediment and runoff reduction under different vegetation cover are still unclear, especially in the eastern Chinese Loess plateau (ECLP). It is necessary to assess the variation of soil erosion and the response of precipitation and vegetation cover to soil erosion on the different slope gradients landform of the CLP.

Therefore, this study selected the hills and gully regions of the ECLP as the study area. According to long time series monitoring data of sediment yields and runoff over three years at three types of slopes and different vegetation cover, we aiming to (1) to compare the effects of vegetation cover in terms of runoff and soil loss in hillslope of the ECLP, and (2) to evaluate the influence of slope characteristics on soil erosion process of the ECLP.

## Materials and Methods

### Experimental sites

**Figure 1:**
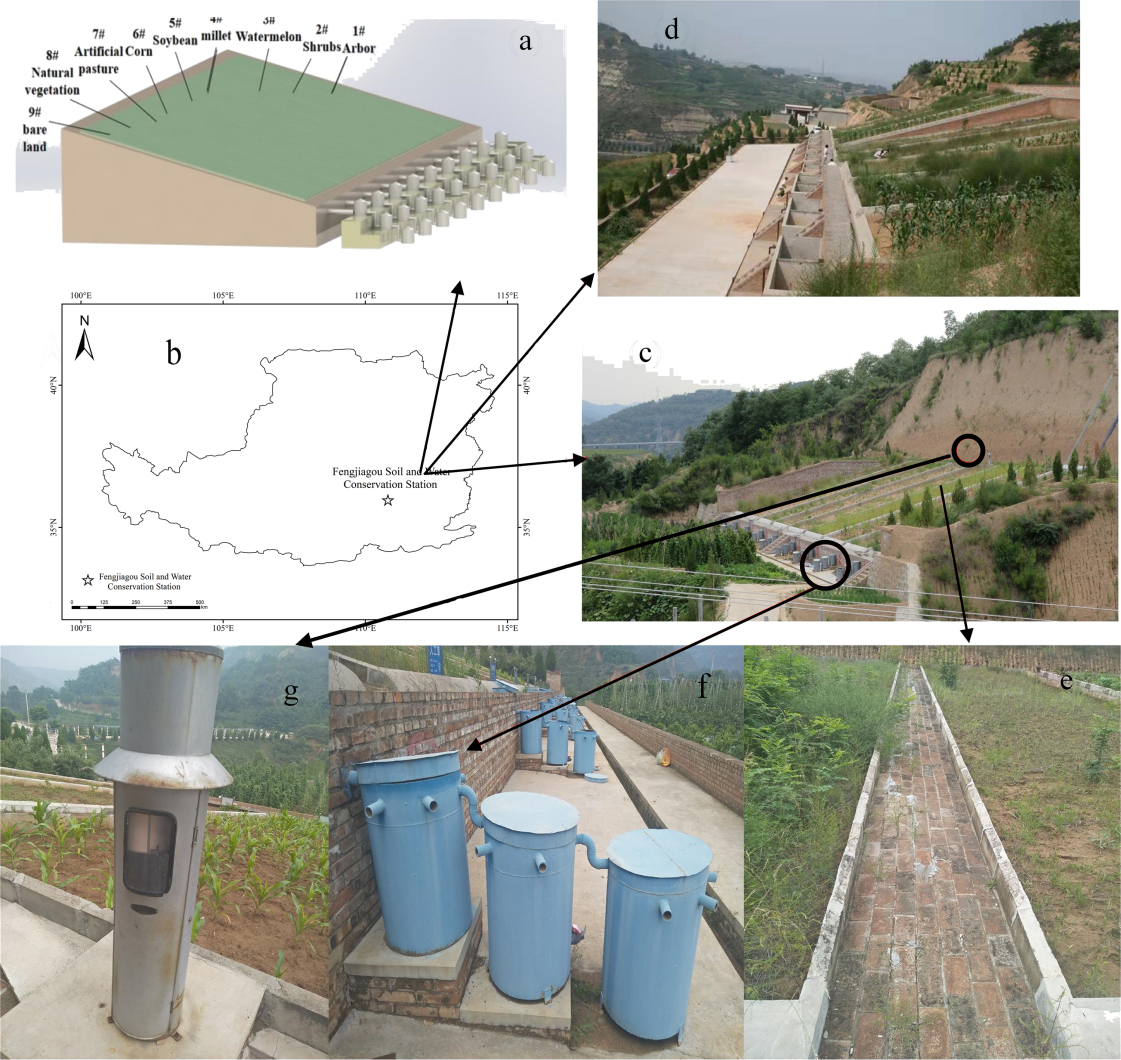
Schematic diagram of the study region and design of experiment. (A) Three-dimensional model about experimental station; (B) location information; (C) erosion plots; (D) erosion plots with different slope; (E) erosion plots interval; (F) runoff and sediment collection device; (G) rain gauge.

The field experiment was carried out at the Fengjiagou observation station of the ECLP (eastern Chinese Losses Plateau, which located the Shanxi province of China) (Fig. 1). This station located at the Ehe river basin (110°48′ to −110°47′E, 36°58′12″ to −35°58′N), a tributary of the Yellow River. It is a typical loess tableland, with a total area of 0.86 km^2^. The topography of the study area is higher in the northwest and lower in the southeast, with many gullies in this region. This region has a classic semi-arid and continental monsoon climate. The annual average temperature here is 9.9°C. The extreme maximum and minimum temperature are 36°C and −18°C, respectively. The annual average sunshine hours are 2,588 h and the frost-free period is 160–200 days. More than 70% of precipitation falls in rainy months (July–September). Reginal annual precipitation amount ranged from 312 mm to 514 mm, with an average of 514 mm. The main soil type in this region is loess according to the Chinese soil classification system in 1995 (Wu et al., 2018). The regional natural vegetation includes *Agropyron mongolicum, Xanthium sibiricum Patrin ex Widde, Setaria viridis (Linn.) Beauv, Amaranthus tricolor, Hetero-pappus altaicus,* etc. The arbor forest of artificial vegetation mainly includes *Robinia pseudoacacia, Platy-cladus orientalis, Populus daviana, Ulmus pumil a and Paulownia fortunei*. The main types of soil erosion include water erosion and gravity erosion.

### Field experiment

Eighteen standard erosion plots of 5 × 20 m with similar aspects and altitudes were built, including nine plots with a 15° incline and covered with different plants. Three land cover types at inclines of 10°, 15°, and 20° were also investigated (Fig. 1D). The boundaries of the runoff plots were fenced (∼50 cm deep) on two sides using cement sheets to ensure that the runoff in the plots did not interfere with the neighboring plots. A storage bucket (of diameter 50 cm and height 60 cm) was installed at the bottom of each plot to collect the runoff and sediment (Fig. 1E). A self-recording siphon rain gauge was installed 50 cm from the cement sheets at the top of the plots to observe rainfall (Fig. 1F). The plots were numbered from 1 to 9 (Fig. 1A) and coved by *arbor* (ARC), *shrub* (SHC), *arable* (ABC), *artificial pasture* (APC), *natural vegetation* (NVC) and *bare land*. Plot 1 is coved by 7-year-old arbor trees, with 15 *Platycladus orientalis planted* (local dominant species, average height is 1.5 m and vegetation coverage is 65%.) Plot 2 is covered by shrub forest (*Forsythia suspensa* and *Syringa* as the dominant species), with 50 trees and an average height of 0.3 m (vegetation coverage is 25%). Plot 3–6 are coved by the local crops (watermelon, millet, soybean, corn). Corn (Plot 6, shown in Fig. 1A) was selected as the ABC type for comparison with other vegetation types, with a total of 50 plants, the average height is 0.7 m, and the vegetation coverage is 25%. According to local custom, corn is sown in early April and harvested in early October every year. According to the standard density of this region, the density of corn is 0.5 plant/m^2^. After harvest, corn stalks are taken away. The plot 7 is covered by artificial grass planting, and its sowing mode is artificial soil preparation and sowing, with an average height of 0.2 m (vegetation coverage is 25%). Plot 8 is abandoned land, and there is no disturbance to the surface after the completion of the plot in 2013. Plot 9 is the control plot (bare land), and the vegetation coverage is 0 (Table 1).

**Table 1:** Vegetation covers of runoff plot in the field experiment.

Runoff plot	Vegetation covers	Dominant species	Vegetation coverage	Mean vegetation height (m)
1#	Arbor	*Platycladu*	65%	3.0
2#	Shrub	*Leptodermis potanin*	25%	0.75
3#	Arable land	*Citrullus lanatus*	25%	0.24
4#				*Triticum aestivum*		1.50
5#				*Glycine max*		0.7
6#		*Zea mays*		1.90
7#	Pasture	*Agropyronmongolicum*	25%	0.3
8#	Natural vegetation	*Ulmus pumila*	–	–
9#	Bare land	–	–	–

To quantitatively analyze the influence of the terrain slope on the sediment yield and runoff, three slope gradient observation sites (10°, 15°, and 20°) with multiple vegetation types (*artificial pasture, natural vegetation*, and *arable land*) were also investigated.

(a: plan of observation area; b: study area location and test design; c: runoff field appearance map of Fengjiagou soil and water conservation station; d: slope gradient observation plots; e: siphon recording rain gauge; f. a bucket used to collect runoff and sediment; g. a sheet between runoff plots.)

### Data collection and analyses

A self-recording siphon rain gauge was used to collect and record the volume and duration, respectively, of each rainfall event. Following each rainfall event, we determined whether the rainfall was erosive by checking whether runoff or sediment had collected in each storage bucket. The rainfall events that produced runoff or sediment in the storage bucket for any runoff plot were defined as erosive rainfall events (Gao et al., 2017). Following each erosive rainfall event, the sediment inside the storage bucket was thoroughly put into glass bottle and the runoff volume was measured using a graduated cylinder. The sediment was separated from the water after settling for 24 h, after which it was dried at 105 °C for 8 h and weighed.

The K-means clustering was used to identify the rainfall regime classification. This method based on a maximum 30 min rainfall intensity (I_30_) and rainfall volume.

## Results

### Rainfall classification

**Figure 2:**
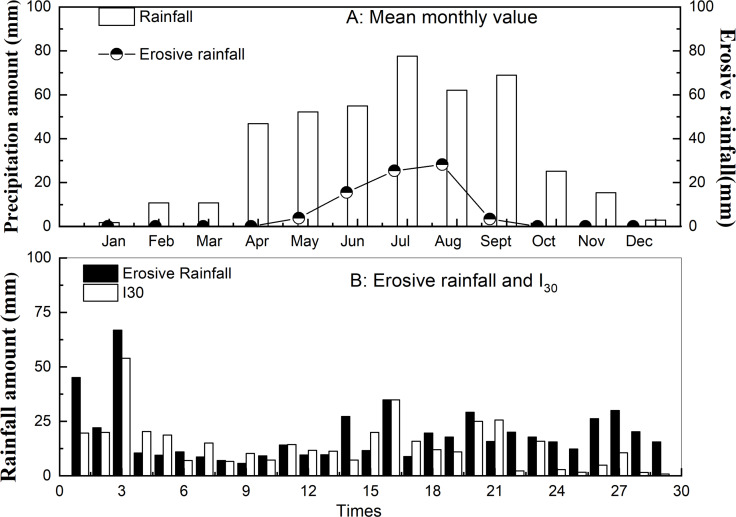
Rainfall characteristics of the small watershed in Fengjiagou of the ECLP. A) Mean monthly rainfall and erosive rainfall value; (B) erosive rainfall and I30 amount of every event.

The eigenvalues of erosive precipitation in a semi-arid environment is among the primary driving and preventive forces to soil erosion (Fu et al., 2011). During the observation period, the mean annual rainfall was 429.3 mm and the mean annual erosive rainfall was 123.5 mm, with large inter-annual variations (Fig. 2). The rainy season lasted from April to September, with 84.45% of the annual rainfall occurring during this period. A total of 29 erosive rainfall events were recorded during 2015-2017. Among these events, 11 (37%) were recorded in July and there was an increasing trend from April to August. The highest volume of erosive rainfall occurred in August, accounting for 72% of the total monthly rainfall. From October to March, almost no erosive rainfall events occurred. In summary, erosive rainfall events were concentrated during the summer, particularly during July and August. The high frequency of erosive precipitation in summer is an important inducing factor of regional soil erosion and flood.

Using K-means clustering, the erosive rainfall events were divided into five classifications: RI (heavy rainfall volumes and moderate rain intensity), RII (small rainfall volumes and small rain intensity), RIII (small rainfall volumes and moderate rain intensity), RIV (moderate rainfall volumes and small rain intensity), RV (moderate rainfall volumes and heavy rain intensity) (Table 2). RI type events had the most rainfall (60.55 mm), followed by RV (36.0 mm), while RII (14.32 mm) and RIII (12.58 mm) type events had relatively low rainfall. Rainfall intensities also differed between the regimes: RV had the highest intensity (33.89 mm h^−1^), followed by RIII (18.3 mm h^−1^), RIV (7.81 mm h^−1^), RI (15.09 mm h^−1^), and RII (7.45 mm h^−1^). Thus, we determined that RI events are characterized by heavy rainfall and moderate intensity, RII events have both low rainfall and intensity, RIII events have moderate rainfall and low intensity, RIV events have moderate rainfall and low intensity, and RV events have moderate rainfall and high intensity. RII events occurred the most frequently (13), followed by RIV (8), RI and RIV (3), and RV (2).

**Table 2:** Statistical features of the different rainfall regimes.

Rainfall regimes	Eigen values	Mean (mm)	Standard deviation	Variation coefficient	Frequency (times)
I	P	60.55	3.83	0.24	3
	I_30_ (mm h^−1^)	15.09	0.36	0.01	
II	P	14.32	5.05	1.78	13
	I_30_ (mm h^−1^)	7.45	2.35	0.74	
III	P	12.58	4.03	1.47	8
	I_30_ (mm h^−1^)	18.3	3.33	0.61	
IV	P	30.27	3.89	0.50	3
	I_30_ (mm h^−1^)	7.81	2.10	0.56	
V	P	36.00	1.1	0.03	2
	I_30_ (mm h^−1^)	33.89	0.98	0.03	

**Notes.**

* P and I_30_ represents precipitation amount and maximum 30-min intensity, respectively.

Erosive rainfall mainly occurred in July and August (Fig. 2), consistent with the semi-arid continental monsoon climate of the ECLP (Zhou et al., 2016). The characteristics of the runoff and sediment yields were strongly influenced by the rainfall type and vegetation cover (Fang et al., 2012). Therefore, understanding the response of runoff and sediment loss for different vegetation types and rainfall regimes is important for controlling soil erosion. Rainfall classifications also play an important role when studying soil erosion.

### Reduction of sediment and runoff under different vegetation types

#### Sediment yield reduction

**Figure 3:**
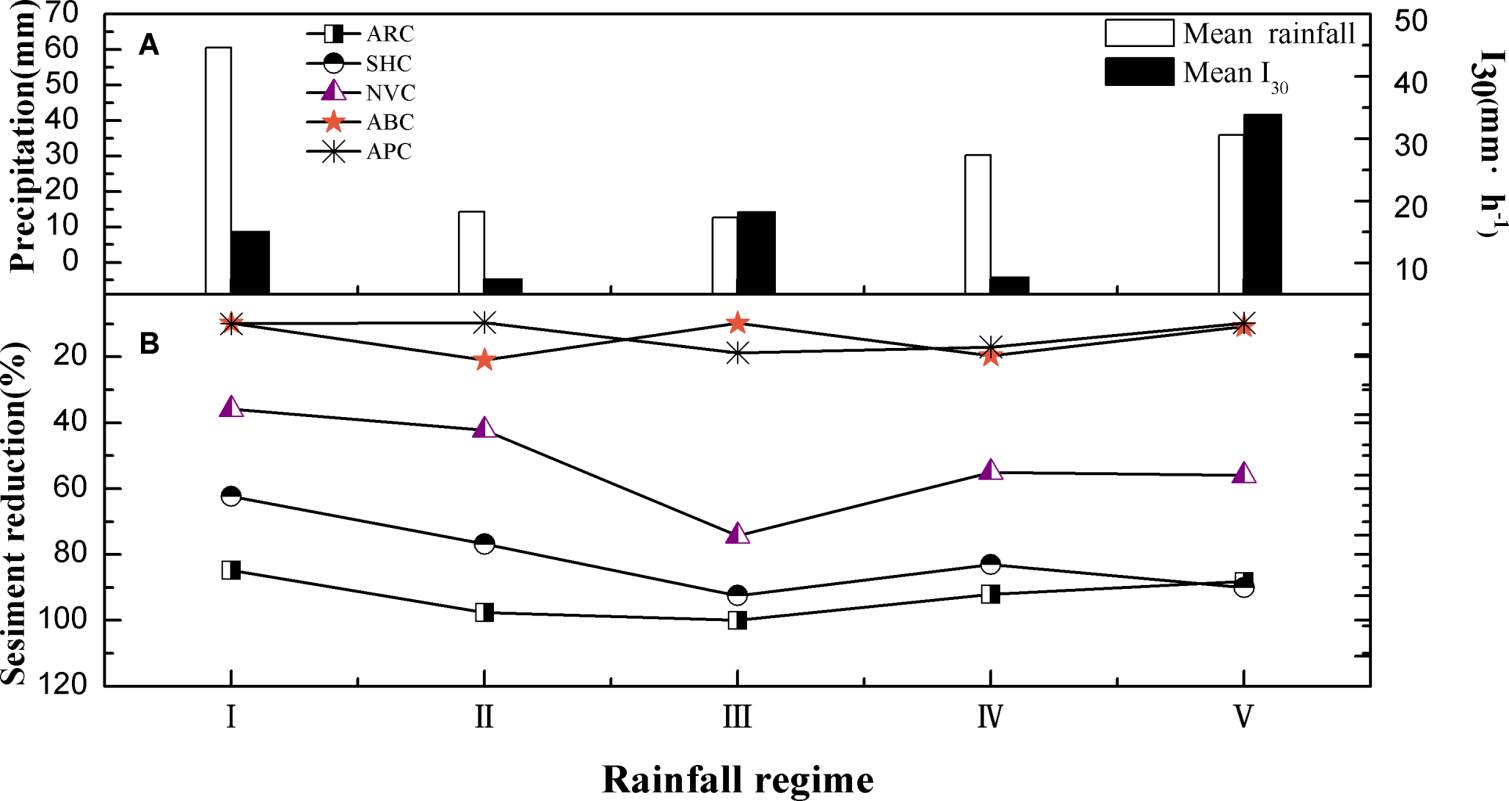
Sediment yield of five different vegetation covers under different rainfall conditions.

Figure 3 shows the variations in sediment yield reduction (expressed as percentages and defined as the ratio of erosion reduction relative to bare land to the amount of sediment in bare land) for five different types of vegetation cover in the different rainfall regimes. For RI (high volume and moderate intensity) rainy type, the highest sediment yield reduction was observed in the ARC plot (84.76%), which was approximately 8.7 and 8.6 times greater than those in the ABC and APC plots, respectively. The SHC plot also shown better sediment yield reduction effect (the sediment yield reduction rate is 62.32%), while the ABC and APC plot almost had no obvious sediment yield reduction effect (less than 10%) in RI type. The sediment yield reduction of all plots under RI displayed an order of the ARC>SHC >NVC >APC>ABC. It is worth noting that the sediment yield reduction of all plots displayed the lowest value under RI. For RII (low volume and intensity), the sediment yield reduction of all plots showed an increased trend with the rainfall intensity and rainfall volume decreased (except the APC plot). The sediment yield reduction in the ARC, SHC, NVC, and ABC plots increased by 12.8%, 14.5%, 6.4%, and 11.22%, respectively. Compared with the five rainfall regimes, almost all plots showed the highest sediment yield reduction under RIII (low volume and moderate intensity), i.e., 100% for ARC, 92.45% for SHC, 74.31% for ABC, and 18.74% for NVC. For RIV, the ARC plot exhibited the highest sediment yield reduction (92%), while the APC plot exhibited the lowest reduction (17%). Under RV (moderate volume and high intensity), the reduction was the highest in the SHC plot (∼90%).

In summary, the ARC plot exhibited the best sediment yield reduction under all the five rainfall regimes, except for RV. However, the sediment yield reduction had a similar trend in the SHC and NVC plots. The performance of the plots is ranked in the order SHC >NVC >APC and ABC, except for RII. The differences in the sediment yield reductions between the APC and ABC plots were minimal for all rainfall regimes. Planting arbor trees and shrubs trees is an effective way to control regional sediment yield.

#### Runoff reduction

**Figure 4:**
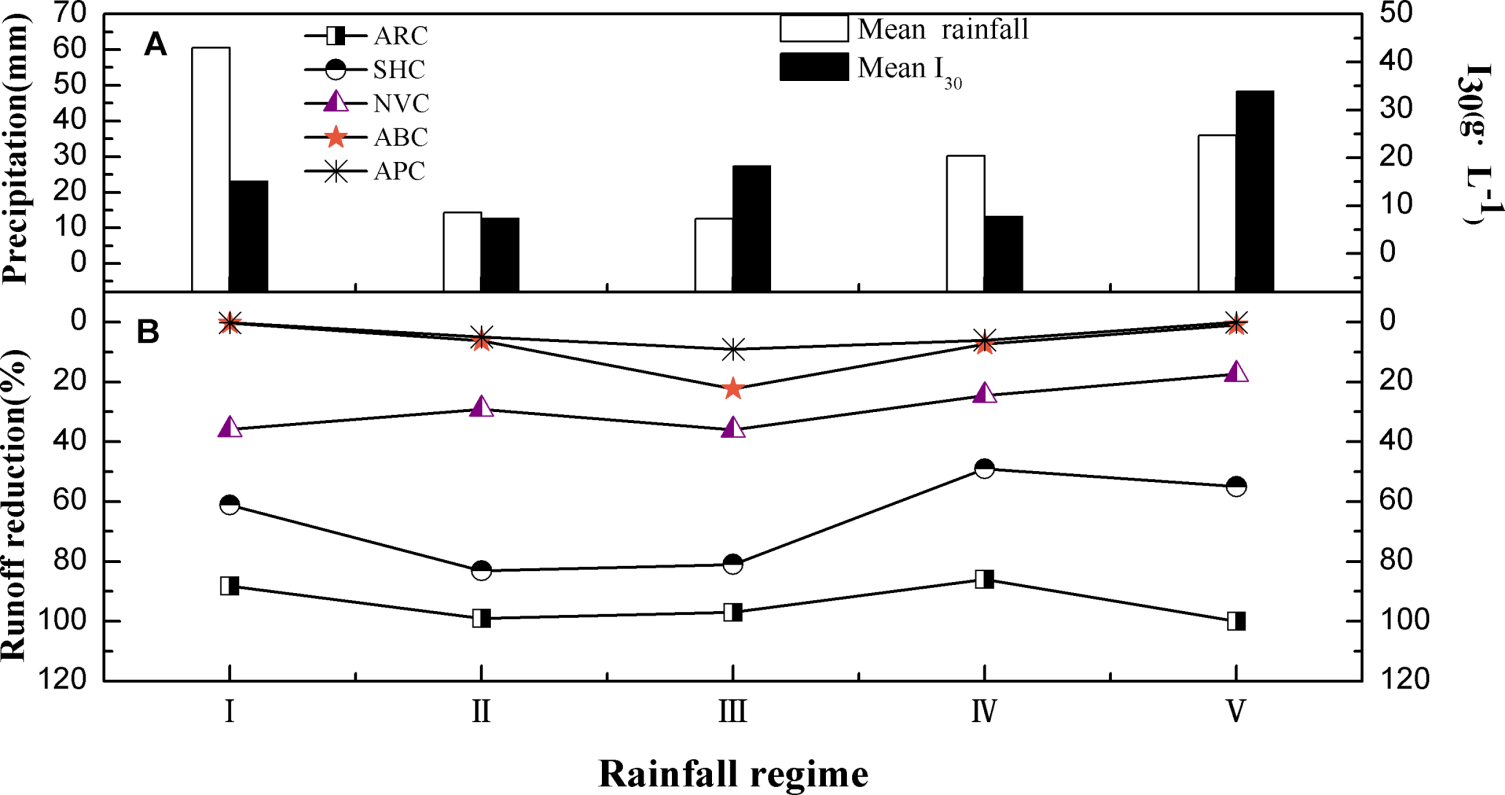
Runoff characteristics of five different vegetation treatments under different rainfall conditions.

Figure 4 shows the reduction (the ratio of runoff reduction relative to bare land to the amount of runoff in bare land) of runoff under five different vegetation cover types for the different rainfall regimes. For all rainfall regimes, the ARC plot had the highest runoff reduction (88.2–100%), followed by the SHC plot (48.99–83.08%). The NVC plot had the lowest runoff reduction (0.12–9.06%).

For the ARC plot, the highest runoff reduction of 100% was observed in RV, while the lowest (88.20%) was observed in RI. The maximum runoff reduction (83.08%) for the SHC plot was observed in RII. On the other hand, the lowest reduction of about 45% was observed in RIII. For all rainfall regimes, the NVC plot exhibited greater reductions in runoff when compared with ABC and PAC plots, while lesser reductions were observed when compared with ARC and SHC plots. The runoff reduction of the ABC and APC plots were generally negligible under all rainfall regimes, except for RIII. Under RIII, the runoff reductions were 24.49% and 17.39% for the ABC and PAC plots, respectively. The ARC plot can prevent more than 80% of the surface runoff for all rainfall regimes, which is of great significance for the prevention of regional soil erosion.

Generally, it can be stated that the ARC plot is the best for reducing runoff, particularly for RV. However, the SHC plot is more effective at reducing runoff when the rainfall volume and intensity is less. Planting arbor trees is still the most effective measure to control surface runoff in study area.

Our rainfall classification results show that events with low volumes and intensities (RII) occurred most frequently, while high-intensity rainfall (RV) and high rainfall volumes (RI) were the least common (Table 1) in this region. Runoff and sediment yields were the highest under RV, followed by RIV (Table 3). Only two RV rainfall events occurred during the monitoring period. Our results indicated the RII (13 times) and RIII (eight times) type are the most threatening erosive rainfall in this region. When planting regional soil and water conservation vegetation, we should consider the vegetation cover types of best reduction sediment and runoff under these rainfall types.

**Table 3:** Characteristics of runoff and sediment yield for different rainfall regimes in the study area.

Rainfall regimes	Mean runoff depth (mm)	Total runoff depth (mm)	Mean sediment yield (g L^−1^)	Total sediment yield (g L^−1^)
I	9.23	27.69	424.1	1,272
II	2.05	24.6	78.0	936
III	5.57	44.56	175.53	1400
IV	3.23	6.46	83.07	166.14
V	12.47	49.88	509.0	2036

### Sediment and runoff yields on different slope gradients

#### Sediment yield

**Figure 5:**
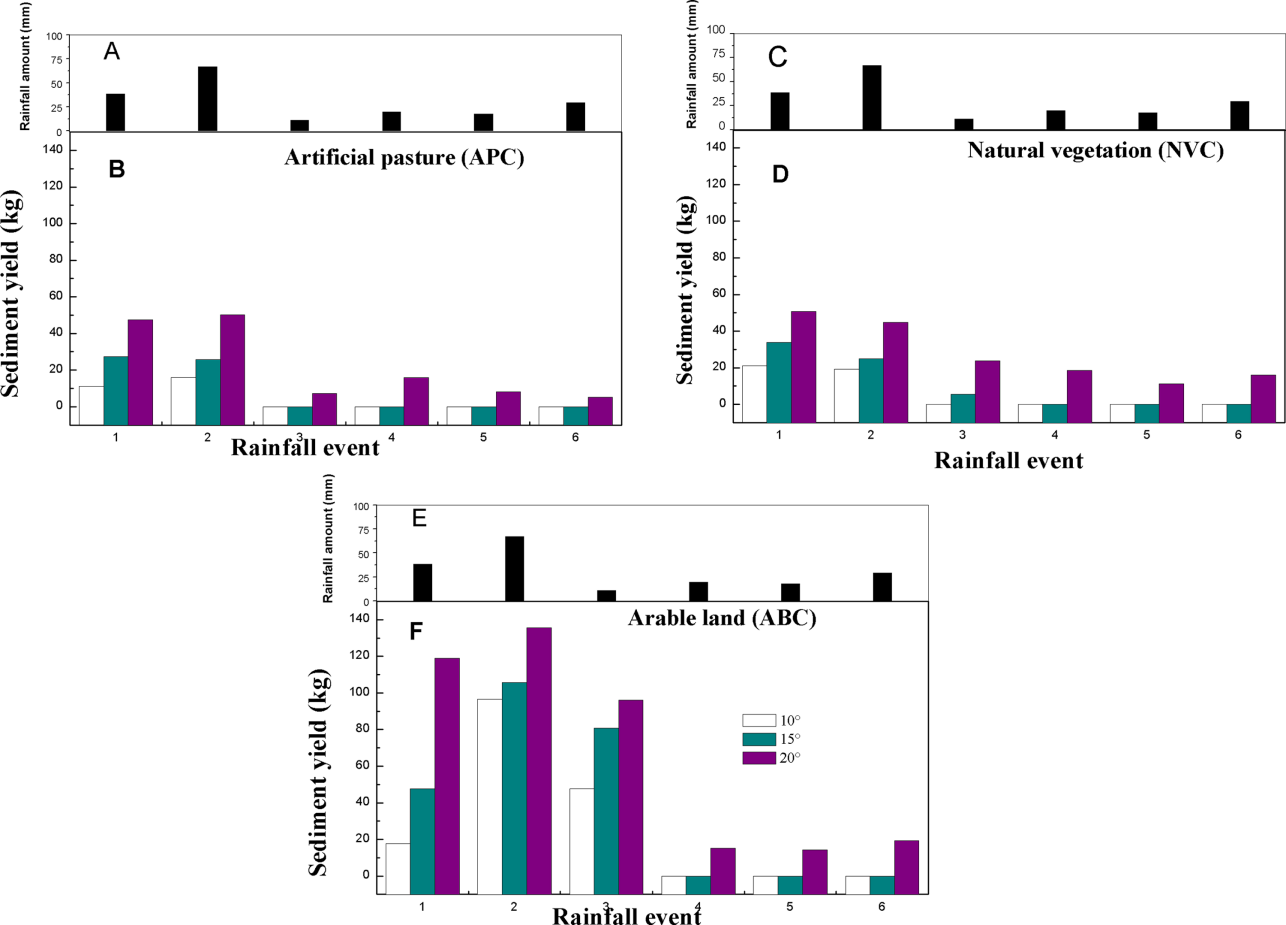
The sediment yield of 3 vegetation treatment in different slope gradients. (A) Rainfall amount of five rainfall event types; (B) sediment yield of APC during five rainfall event types; (C) rainfall amount of five rainfall event types; (D) sediment yield of NVC during five rainfall event types; (E) rainfall amount of five rainfall event types; (F) sediment yield of ABC during five rainfall event types.

Figure 5 shows the variations in sediment yield among the three vegetation covers and slope gradients following six erosive rainfall events. Erosive rainfall events were occurred on June 30 (named as “1”, belong to RI), August 18 (named as “2”, belong to RV), September 5 (named as “3”, belong to RIII), August 10 (named as “4”, belong to RII), August 20 (named as “5”, belong to RII), and August 26 (named as “6”, belong to RIV). Compared with the three vegetation covers, the sediment yield of APC and NVC did not show significant variation during six erosive events, while the sediment yield of ABC displayed an obvious variation during six erosive. Sediment yields ranged from 0–50.88 kg/m^3^ for the NVC plot, 0–47.5 kg/m^3^ for the APC plot, and 19.67–135.63 kg/m^3^ for the ABC plot. The ABC plot had the highest sediment yield. There was a remarkable increase in the sediment yield from all plots as the slope gradient increased. The maximum sediment yields from the APC (50.3 kg/m^3^) and ABC (135.63 kg/m^3^) plots occurred during the second erosive rainfall event (RV type), while the maximum sediment yield from the NVC plot (50.88 kg/m^3^) occurred during the first erosive rainfall event (RI type). The fourth, fifth, and sixth erosive rainfall events did not produce significant sediment yields from any of the plots with slopes of 10° or 15°. Sediment yields from the APC and NVC plots with 20° slopes were considerably higher compared to those with 10° and 15° slopes, while there were only slight variations in sediment yield between plots with 10° and 15° slopes. Hence, arable land should be a focal point when implementing erosion reduction management strategies in this region. Local crops should be preferentially distributed in the farmland with slopes less than 15°, which can effectively reduce regional soil erosion.

#### Runoff yield

**Figure 6:**
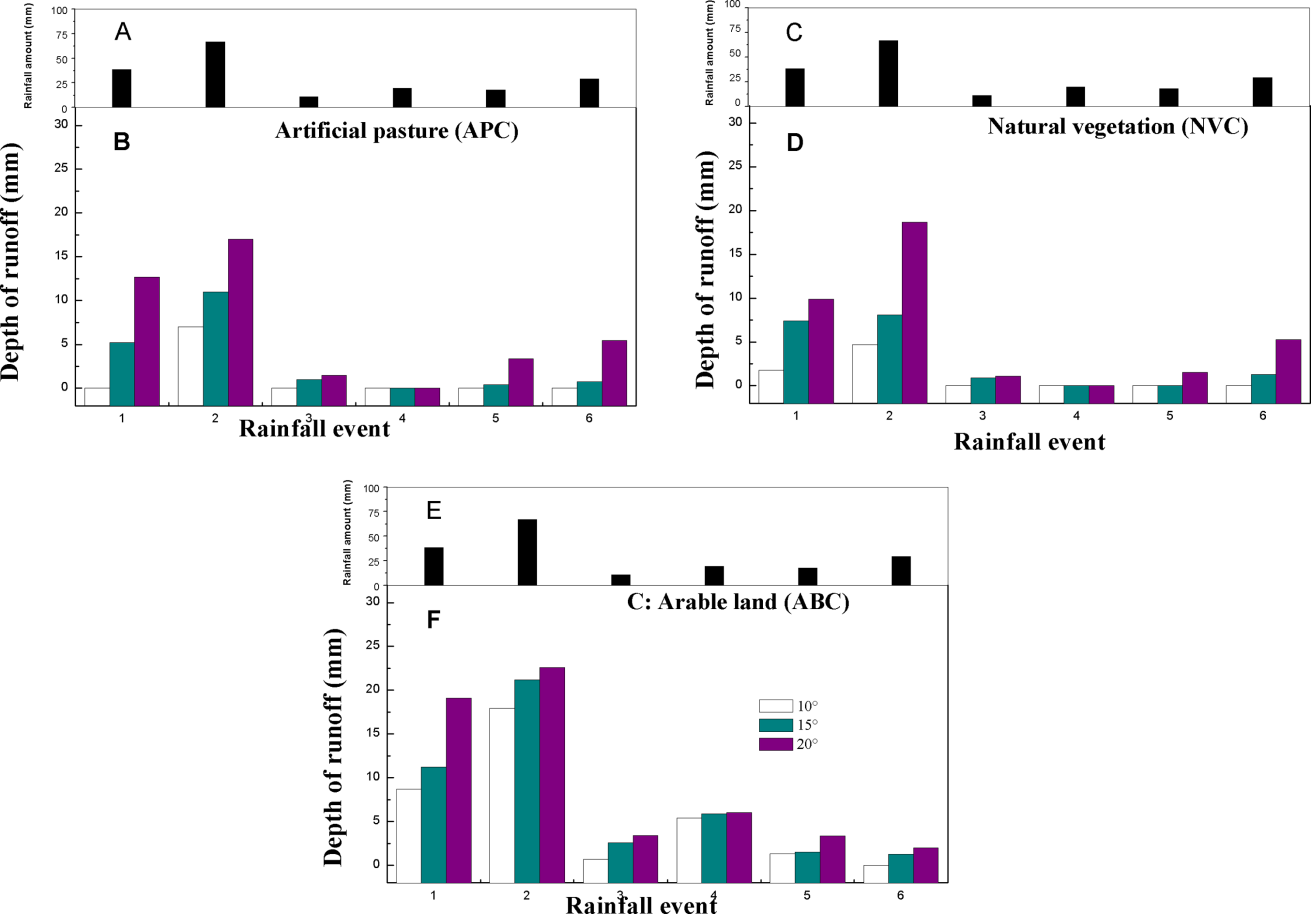
The runoff depth of three vegetation treatment in different slope gradients. (A) Rainfall amount of five rainfall event types; (B) sediment yield of APC during five rainfall event types; (C) rainfall amount of five rainfall event types; (D) sediment yield of NVC during five rainfall event types; (E) rainfall amount of five rainfall event types; (F) sediment yield of ABC during five rainfall event types.

Variations in runoff yield from the plots with vegetation cover under different slope gradients are shown in Fig. 6. A positive relationship between runoff depth and slope gradient was observed for all the vegetation types. Runoff yields from all plots with 20° slopes were considerably higher compared to those with 10° and 15° slopes. When the rainfall is light (like erosive rainfall events 3, 4 and 5), the variation of runoff yield among three slopes is small. However, the difference of runoff yield among three slope plots is remarkable when the rainfall is higher than the moderate rainfall amount. For the APC plot, the highest runoff yields (7, 11, and 17 mm for 10°, 15°, and 20° slopes, respectively) were observed for all the slopes following the second erosive rainfall event. With the exception of the first and second rainfall events, runoff was rarely observed on a 10° slope. During the second erosive events (RV type), there were a significant increase in runoff yield with slope gradients, especially in the NVC plots (4.7, 8.1, and 18.7 mm for 10°, 15°, and 20° slopes, respectively). Compared to the APC and NVC plots, runoff from the ABC plot was always higher, ranging from 0.7–22.6 mm. In summary, the influence of slope grade on runoff yield is more obviously during larger and moderate rainfall, especially in the APC and NVC plots. In order to better control the runoff yield reduction, the distribution of local cultivated land should not exceed 10° slope.

## Discussion

### Responses of runoff and sediment yield to vegetation type

Vegetation cover plays a critical role in regional soil erosion processes (Zhou, Shangguan & Zhao, 2006). Different vegetation types have various impacts on soil and water conservation due to variations in the vegetation coverage (Andreu et al., 1998) and root biomass density (Fattet et al., 2011). Among the rates of runoff and sediment loss for the five vegetation types analyzed in this study, we found that the ARC type was most beneficial for soil and water conservation in the ECLP, in agreement with the findings of Feng et al. (2017). The benefits of ARC in soil and water conservation can be attributed to a large canopy, elevated above- and below-ground biomass, and litter-fall mass (Xu et al., 2012). Larger tree canopies can effectively block and reduce the erosion by rainfall, and can reduce the generation of surface runoff. At the same time, ARC usually has more developed roots that can fix the soil on different slopes, thus further reducing soil migration with runoff. In addition, a large number of falling objects (dead branches and fallen leaves) produce a rough surface underneath this vegetation type. Thus, ARC’s soil and water conservation effects are superior. The strongest soil conservation capacity of the shrub cover had also been exported in other regions of the CLP, such as in the Yangjungou Catchment (Zhou et al., 2016) and Southern Shaanxi Province (Zhou et al., 2016). Besides, the SHC type was also relatively beneficial for soil and water conservation, which can be attributed to a high vegetation density and high coverage (Hao et al., 2016). High density growth in the SHC plots had a weakening effect on regional erosive precipitation, which can effectively reduce runoff and sediment yields.

The NVC type is considered as the best vegetation cover type for soil and water conservation by local government administrators. However, our results indicated the effect of soil and water conservation of the NVC is weaker than that of the ARC and SHC, especially in the ECLP. For the NVC type, a soil crust forms when the vegetation is harvested (Stavi et al., 2009; Sun, Kang & Jiang, 2010), which partially protects the soil surface and reduces sediment yield but increases the runoff. Consequently, NVC was more effective for sediment retention (sediment yield reduction ranged from 35.82% to 74.31%) compared to runoff reduction (runoff yield reduction rate ranged from 17.39% to 39.06%). Both APC and ABC types have limited benefits for controlling regional soil erosion because farming practices disturb the soil surface (Bakker, Govers & Rounsevell, 2004; El Kateb et al., 2013). Thus, ABC and APC types are not recommended for managing regional soil erosion due to low coverage (Andreu et al., 1998) and root biomass density (Fattet et al., 2011). This finding is inconsistent with that of Lin et al. (2019), who reported that wheat cultivation had a certain influence on controlling regional soil erosion.

Crop species and meteorological characteristics in the local region should be noted when developing regional soil and water conservation policies. For example, when the rainfall volume is high, runoff and soil yields from the ABC and APC plots tended to be low, which is consistent with the findings of Gao et al. (2017) for the rocky mountain areas of the Taihang Mountains. When rainfall was the most intense, the SHC type was the most effective in reducing soil erosion, while the ARC type was the most effective in reducing runoff. Thus, soil loss under the ARC type was sensitive to the rainfall regime. The effectiveness of the SHC type at reducing sediment loss during high intensity rainfall events was also confirmed by Peng & Wang (2012) on the karst slopes of southwestern China.

Similar studies have been conducted in other parts of China (Table 4). Most studies show that in different regions of China, except karst regions, the most frequent rainfall regime is low amount and intensity, while serious soil and water losses throughout the year are often caused by rain events with more frequency. In different regions, the types of vegetation available for soil and water conservation may vary due to differences in soil properties and climatic conditions (Albaugh, Dye & King, 2013). In the Red earths regions of China, studies have noted that *Pinus massoniana* secondary forests and natural succession type forests are most suitable for regional water and soil conservation. In the purple soil regions of China (Wang, Zhu & Wu, 2017), bamboo forests are the most effective in preventing and controlling soil erosion. In the karst slopes of China (Peng & Wang, 2012), forested land is considered to be the best vegetation type for soil erosion control. In the rocky mountain area of Taihang, researchers have found that trees are the best vegetation type for soil erosion control (Gao, Rao & Cui, 2017). In summary, the protective effect of tall arbors on controlling regional soil erosion has been verified in different regions, which is consistent with the results of this study.

**Table 4:** The information of rainfall types and soil and water conservation plant types in different study areas.

Study area	The most frequent erosion rainfall	Rainfall regime of water and soil loss	Vegetation of soil and water conservation
Red earths region of China	Low intensity and short duration	Moderate rainfall and intensity	*Pinus massoniana* secondary forest and natural succession type forest
Purple soils region of China	Low intensity and long duration	High intensity and short duration	Bamboo forest land
Karst slopes of China	High intensity and rainfall	Extreme rainfall storm	Forestland.
Rocky mountain area of Taihang	Low rainfall and intensity		Arbor

The RII and RIII as the common erosion rainfall for the ECLP, should be paid more attention when developing regional soil and water conservation policies. According to our research, the ARC and SHC have significant effect of reduction runoff and sediment under these two rainfall types. Therefore, the ARC and SHC are the best vegetation cover type for soil and water conservation of the ECLP.

### Runoff and sediment response to vegetation type and slope gradient

The slope gradient is a major driver for regional soil erosion, particularly during high intensity rainfall events (Chen et al., 2011; Sirjani & Mahmoodabadi, 2014). In this study, the impact of slope gradient on runoff and sediment yield was apparent. For example, runoff and sediment yields from a slope gradient of 20° were remarkably higher than those from a gradient of 10° or 15° for all three vegetation types (Figs. 5 and 6). These results are consistent with the results for the ABC vegetation type observed by Huang, Fu & Chen (2005), who determined that farmlands are vulnerable to soil erosion when the slope gradient exceeds 10°. This can be attributed to human activities (e.g., soil tillage) which result in more soil loss and surface runoff (El Kateb et al., 2013). The influence of the slope gradient on runoff and sediment yields had remarkable temporal variations. For example, rainfall events with the highest volumes and intensities produced the largest runoff and sediment loss for all three vegetation types. The APC and NVC types exhibited high levels of runoff and sediment loss from slope gradients of 10° and 15° when the first and second rainfall events (more volume and higher intensity) occurred. From the fourth to sixth rainfall events, the influence of the slope gradient on soil erosion gradually weakened. Thus, the combined effects of slope gradient and rainfall characteristics (amount, duration, and intensity) on runoff and sediment yields warrant further exploration and ongoing data collection (Deng et al., 2019).

## Conclusions

Runoff and sediment loss were evaluated considering different vegetation cover types, slope gradients, and rainfall characteristics. Using three years of observational data from field experiments at the Fengjiagou water and soil conservation station, this study investigated the response of runoff and sediment yields to various vegetation types and slope gradients under erosive rainfall in hills and gully areas of the ECLP. We draw the following conclusions:

(1) In the ECLP, the rainy season lasted from April to September and erosive rainfall was most likely to occur during July and August. The RII (13 times) and RIII (8 times) type are the most threatening erosive rainfall in this region. When planting regional soil and water conservation vegetation, we should consider the vegetation cover types of best reduction sediment and runoff under these rainfall types.
(2) Overall, the ARC and SHC type were most beneficial for soil and water conservation in the ECLP. The NVC was more effective for sediment yield reduction. While, the APC and ABC are not conductive to the prevention of regional soil erosion.
(3) Runoff and sediment yields increased with the slope gradient. The influence of slope grade on soil erosion is more obvious during larger and moderate rainfall. Farmland was vulnerable to soil erosion when the slope gradient exceeded 10° .

##  Supplemental Information

10.7717/peerj.11226/supp-1 Data S1 Raw data

## Additional Information and Declarations

### Data Availability

The following information was supplied regarding data availability: The raw measurements are available in the Supplemental File.
